# The synthetic opioid fentanyl enhances viral replication *in vitro*

**DOI:** 10.1371/journal.pone.0249581

**Published:** 2021-04-14

**Authors:** Ling Kong, Rebekah Karns, Mohamed Tarek M. Shata, Jennifer L. Brown, Michael S. Lyons, Kenneth E. Sherman, Jason T. Blackard

**Affiliations:** 1 Division of Digestive Diseases, Department of Internal Medicine, University of Cincinnati College of Medicine, Cincinnati, OH, United States of America; 2 Digestive Health Center, Cincinnati Children’s Hospital, Cincinnati, OH, United States of America; 3 Addiction Sciences Division, Department of Psychiatry and Behavioral Neuroscience, University of Cincinnati College of Medicine, Cincinnati, OH, United States of America; 4 Center for Addiction Research, University of Cincinnati College of Medicine, Cincinnati, OH, United States of America; 5 Department of Emergency Medicine, University of Cincinnati College of Medicine, Cincinnati, OH, United States of America; National Institute of Infectious Diseases, JAPAN

## Abstract

The US is in the midst of a major drug epidemic fueled in large part by the widespread recreational use of synthetic opioids such as fentanyl. Persons with opioid use disorder are at significant risk for transmission of injection-associated infections such as hepatitis B virus (HBV) and hepatitis C virus (HCV). Commonly abused substances may antagonize immune responses and promote viral replication. However, the impact of synthetic opioids on virus replication has not been well explored. Thus, we evaluated the impact of fentanyl and carfentanil using *in vitro* systems that replicate infectious viruses. Fentanyl was used in cell lines replicating HBV or HCV at concentrations of 1 ng, 100 ng, and 10 ug. Viral protein synthesis was quantified by ELISA, while apoptosis and cell death were measured by M30 or MTT assays, respectively. HCV replicative fitness was evaluated in a luciferase-based system. RNAseq was performed to evaluate cellular gene regulation in the presence of fentanyl. Low dose fentanyl had no impact on HCV replication in Huh7.5JFH1 hepatocytes; however, higher doses significantly enhanced HCV replication. Similarly, a dose-dependent increase in HCV replicative fitness was observed in the presence of fentanyl. In the HepG2.2.15 hepatocyte cell line, fentanyl caused a dose-dependent increase in HBV replication, although only a higher doses than for HCV. Addition of fentanyl resulted in significant apoptosis in both hepatocyte cell lines. Cell death was minimal at low drug concentrations. RNAseq identified a number of hepatocyte genes that were differentially regulated by fentanyl, including those related to apoptosis, the antiviral / interferon response, chemokine signaling, and NFκB signaling. Collectively, these data suggest that synthetic opioids promote viral replication but may have distinct effects depending on the drug dose and the viral target. As higher viral loads are associated with pathogenesis and virus transmission, additional research is essential to an enhanced understanding of opioid-virus pathogenesis and for the development of new and optimized treatment strategies.

## Introduction

The US is experience a major opioid crisis [1], and the harmful effects of opioid use disorder (OUD) continue unabated despite the availability of harm reduction and prevention strategies. From 2013 to 2019, the number of synthetic opioid-involved deaths increased over 1,000% from 3,105 to 36,359 [2]. Recent data demonstrate that approximately 90% of unintentional overdoses involved fentanyl or fentanyl analogs [3–6].

Opioids comprise both endogenous and exogenous or synthetic compounds that function by activating several opioid receptors. Opioid receptors are expressed on a variety of immune cells including lymphocytes, monocytes, macrophages, and neutrophils [7–9]. They are also expressed in the liver–including in hepatocytes and hepatic stellate cells–and are important mediators of liver disease progression [10–16]. Among persons with OUD, there is considerable risk of transmitting viral infections such as the human immunodeficiency virus (HIV), hepatitis B virus (HBV), and hepatitis C virus (HCV). An association between HIV infection and fentanyl use has been reported in several settings [17, 18]. Drugs of abuse are known to promote viral replication and virus-mediated pathology (reviewed in [8, 19]). Nonetheless, to date, there are no published reports on the effects of fentanyl, fentanyl analogs, or fentanyl metabolites on virus replication despite their widespread recreational use. Because a robust understanding of opioid-host and opioid-virus interactions is essential for the development of new and optimized treatment and harm reduction strategies, we evaluated the impact of fentanyl on hepatic viral replication *in vitro*.

## Methods

### Cell lines and reagents

HepG2.2.15 cell line contains two copies of stably integrated HBV and produces high levels of hepatitis B e antigen and hepatitis B surface antigen (HBsAg) [20]. The Huh7.5_JFH1_ cell line–which produces infectious HCV genotype 2a virions–was provided by Dr. Guangxiang Luo [21] and maintained in DMEM high glucose medium supplemented with 10% FBS, penicillin (100 U/mL), and streptomycin (100 mg/mL). Huh7.5 cells were provided by Apath LLC (St. Louis, MO). SH-SY5Y is a human neuroblastoma cell line with robust expression of the mu opioid receptor [22, 23] and was obtained from ATCC. For experiments with infectious HCV, virions were harvested from the supernatants of Huh7.5_JFH1_ cells, filtered, spun at high speed to pellet cellular debris, and stored at −80°C prior to use as described previously [24]. Naïve Huh7.5 cells were infected for 1 hour with 0.5 TCID_50_ of virus. Virus was then removed, and cells were washed with PBS multiple times to remove unbound virus prior to drug exposure.

Fentanyl and carfentanil oxalate were obtained from Cerilliant (Round Rock, TX). Per the manufacturer’s certificate of analysis, these reagents are suitable for the *in vitro* identification, calibration, and quantification of analytes in analytical and R&D applications. Fentanyl represents a μ-opioid receptor agonist that was approved in the US as an intravenous anesthetic in 1972 and is approximately 50–100 times more potent than morphine [25]. Carfentanil is a synthetic fentanyl analogue approved for veterinary use with an analgesic potency that is 20–30 times that of fentanyl and approximately 10,000 times that of morphine [26]. Fentanyl and carfentanil are metabolized by the CYP3A4 pathway in the liver.

### Mu opioid receptor quantification

Mu opioid receptor expression was quantified in several hepatocyte-derived cell lines, as well as the SH-SY5Y neuroblastoma cell line as a positive control. 2 x 10^6^ cells were resuspended in lysis buffer and diluted 1:2. 100 uL of cell/lysis suspension was used in the human opioid receptor mu 1 (OPRM1) ELISA (MyBioSource; San Diego, CA).

### Viral protein quantification

HBV HBsAg protein was measured in culture supernatants by the QuickTiter Hepatitis B Core Antigen ELISA Kit (Cell Biolabs, Inc.; San Diego, CA) with a lower limit of detection (LLD) of 1 ng/mL. HCV core protein was quantified in cell culture supernatants using the QuickTiter Hepatitis C Core Antigen ELISA Kit (Cell Biolabs, Inc.; San Diego, CA) with an LLD of 1 ng/mL.

### Drug exposure

The Huh7.5_JFH1_ and HepG2.2.15 cell lines were seeded at 500,000 cells per well. Fentanyl or carfentanil was added to culture medium after 24 hours. After 24 hours of incubation with drug, HCV core (ng/mL), or HBsAg (ng/mL) was quantified in culture supernatants.

### Cell viability

500,000 cells were seeded per well in 96-well format. Drug was added to culture medium after 24 hours. After 24 hours of incubation with drug, the potential toxicity was evaluated by the MTT Cell Proliferation Assay Kit (Abcam; Cambridge, MA).

### HCV replicative fitness

HCV replicative fitness was measuring using patient-derived NS5B sequences inserted into an NS5B shuttle vector. As reported previously, the shuttle vector included the non-structural genes NS3, NS4A, NS5B and the 3′ untranslated region (UTR) derived from the genotype 1a strain H77, as well as several cell culture adapting mutations and a firefly luciferase reporter gene [27]. Full-length NS5B sequences were amplified from two patients with chronic HCV, and patient-specific plasmids and RNA were synthesized and used for transient replication assays in Huh7 hepatocytes. Transfected cells were plated in 96-well plates. At 4 hours post-transfection, the wells from one plate were harvested for luciferase quantification to normalize for transfection efficiency. The remaining plate was incubated at 37°C for 4 days, and luciferase activity was quantified to measure patient-specific replicative fitness.

### Hepatic apoptosis

Cells were seeded at 500,000 per well. Fentanyl was added to culture medium after 24 hours. After 3 days of incubation with drug, M30 (units/mL) was quantified in culture supernatants using the M30 Apoptosense ELISA (TECOmedical Group) with an LLD of 20 U/L.

### RNAseq analysis

The NEBNext Poly (A) mRNA Magnetic Isolation Module (New England Biolabs, Ipswich, MA) was utilized for polyA RNA purification with a total of 300 ng total RNA as input. A PrepX mRNA Library kit (WaferGen) combined with Apollo 324 NGS automated library prep system was used for library preparation. Sample-specific indices were added to each adaptor-ligated cDNA sample, using the universal and index-specific primer with a limited PCR cycle number, and the amplified library was cleaned by AMPure XP beads in the Apollo 324 system with a final elution volume of 16 μl. To confirm the quality and yield of the purified library, 1 μl was analyzed by Bioanalyzer (Agilent, Santa Clara, CA) using a DNA high sensitivity chip. To accurately quantify the library concentration for clustering, the library was diluted 1:10^4^ in dilution buffer (10 mM Tris-HCl, pH 8.0 with 0.05% Tween 20), and qPCR was performed with the Kapa Library Quantification kit (Kapabiosystem, Woburn, MA) using ABI’s 9700HT real-time PCR system (Thermo Fisher). Individually indexed and compatible libraries were proportionally pooled (~25 million reads per sample in general) for clustering in cBot system (Illumina, San Diego, CA). Libraries at a final concentration of 15 pM were clustered onto a single read flow cell using the Illumina TruSeq SR Cluster kit v3, and sequenced to 50 bp using the TruSeq SBS kit and the Illumina HiSeq system.

Following the removal of primers and barcodes, raw reads were aligned to the Hg38 genome using Kallisto, which employs pseudoalignment to rapidly and accurately determine compatibility of reads with genomic targets. Annotations were provided by UCSC, with output of transcripts per million (TPM). Raw data were log_2_-tranformed, normalized with a 75^th^ percentile shift, and baselined to the median of all samples. Further, transcripts were filtered to include only those with TPM > 3 in 100% of samples in at least one experimental condition (N = 11,891). Differential expression was assessed using moderated t-tests with a cutoff of p < 0.05 and fold change > 1.25. Transcripts meeting significance criteria were submitted to ToppGene and ToppCluster for ontological analyses.

Candidate gene sets were generated using Gataca (https://gataca.cchmc.org/gataca/) and ToppGene (https://toppgene.cchmc.org) and submitted to principal component analysis, which creates comprehensive variables through linear combinations of numerous related variables. Thus, expression of multiple candidate genes was distilled into principal components. Samples were plotted according to values of principal components 1 and 2 to determine sample distribution according to candidate gene expression. RNAseq data are available through GEO using accession number GSE167922.

### Statistical analysis

Technical duplicates were performed for each experimental condition, and data were converted to fold change from the “no drug” condition with error bars representing the standard deviation of the duplicate measures. ANOVA was used to evaluate statistical significance (p < 0.05) compared to the “no drug” condition.

## Results

### Hepatocyte cells lines expression mu opioid receptor

**Fig 1:**
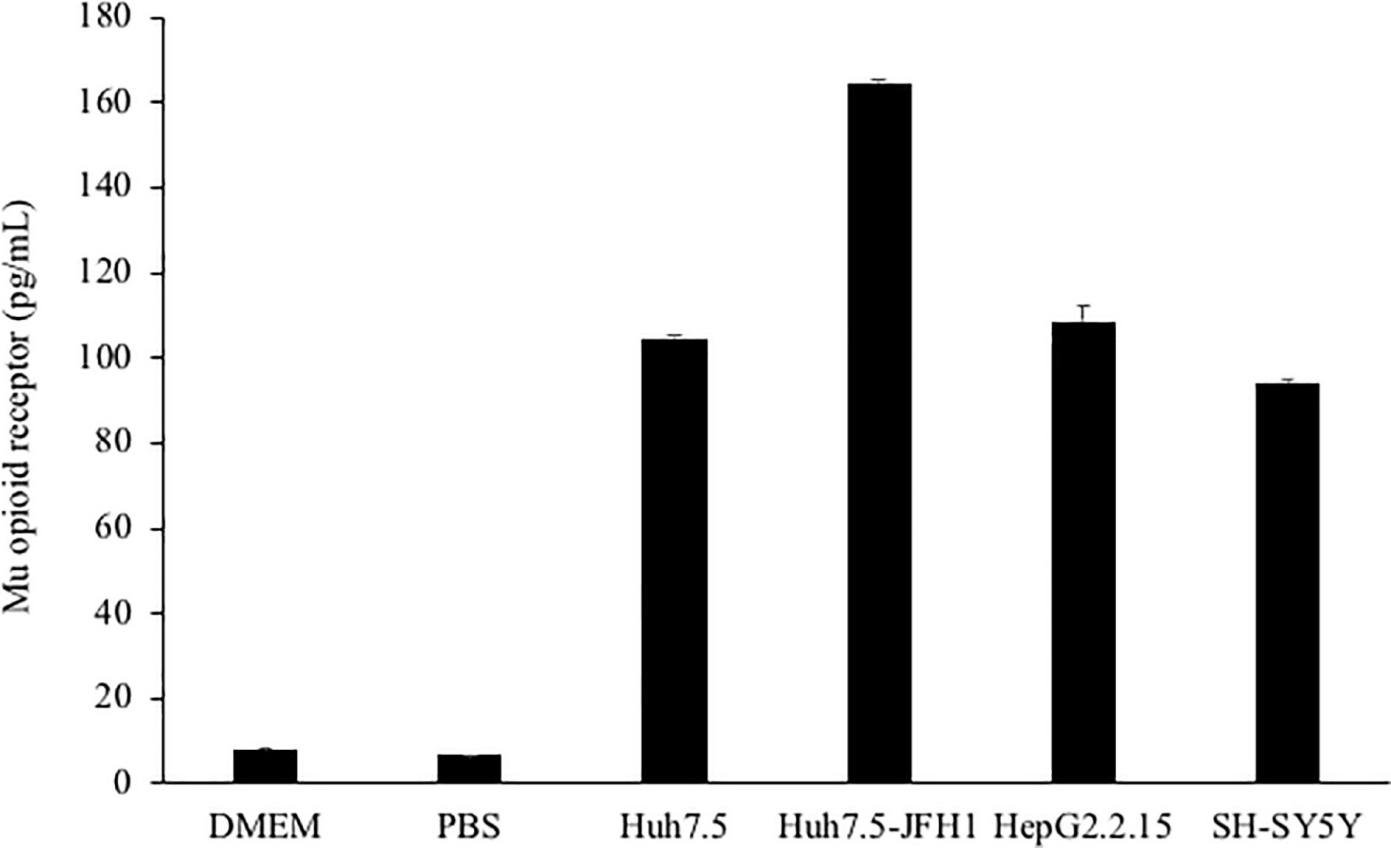
Mu opioid receptor expression was quantified ELISA in 2 x 10^6^ cells resuspended in lysis buffer and diluted 1:2. DMEM = Dulbecco’s Modified Eagle Medium (DMEM); PBS = phosphate buffered saline.

Mu opioid receptor expression was quantified by ELISA in several hepatocyte-derived cell lines, including Huh7.5_JFH1_ (HCV-infected), the parental / uninfected Huh7.5, and HepG2.2.15 (HBV-infected). The SH-SY5Y neuroblastoma cell line was included as a positive control. As shown in Fig 1, all hepatocyte-derived cell lines expresses mu opioid receptor at or above the levels present in the SH-SY5Y cell line. Mu opioid receptor expression was minimal in DMEM and PBS, indicating minimal background signal.

### Opioids enhance viral replication

**Fig 2:**
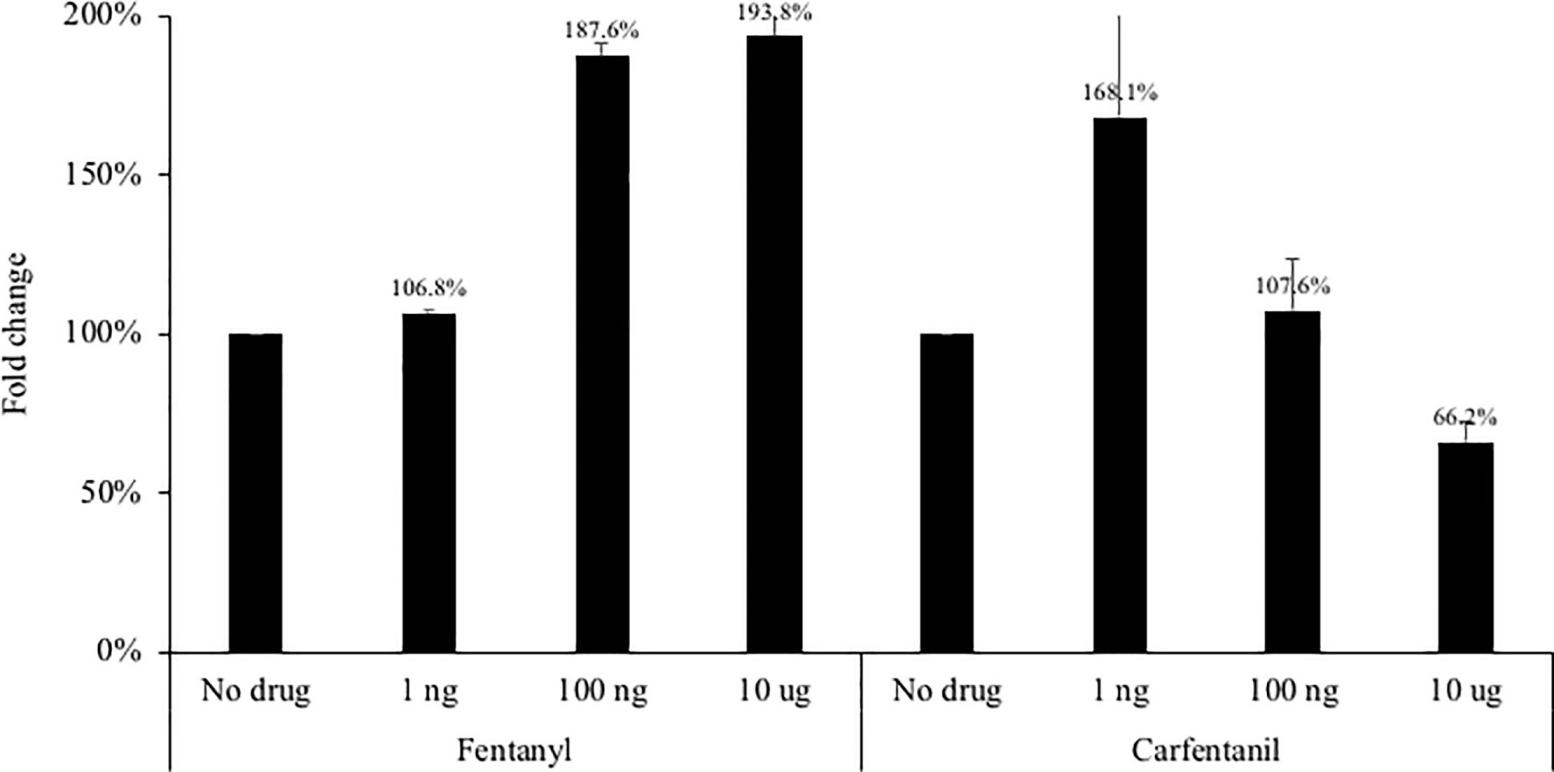
The Huh7.5JFH1 hepatocyte cell line–which constitutively expresses infectious HCV–was seeded at 500,000 cells per well. Fentanyl or carfentanil was added to culture medium after 24 hours. After 24 hours of incubation with drug, HCV core protein (ng/mL) was quantified in culture supernatants. ANOVA for dose effect: p = 0.0007 for fentanyl and p = 0.041 for carfentanil.

**Fig 3:**
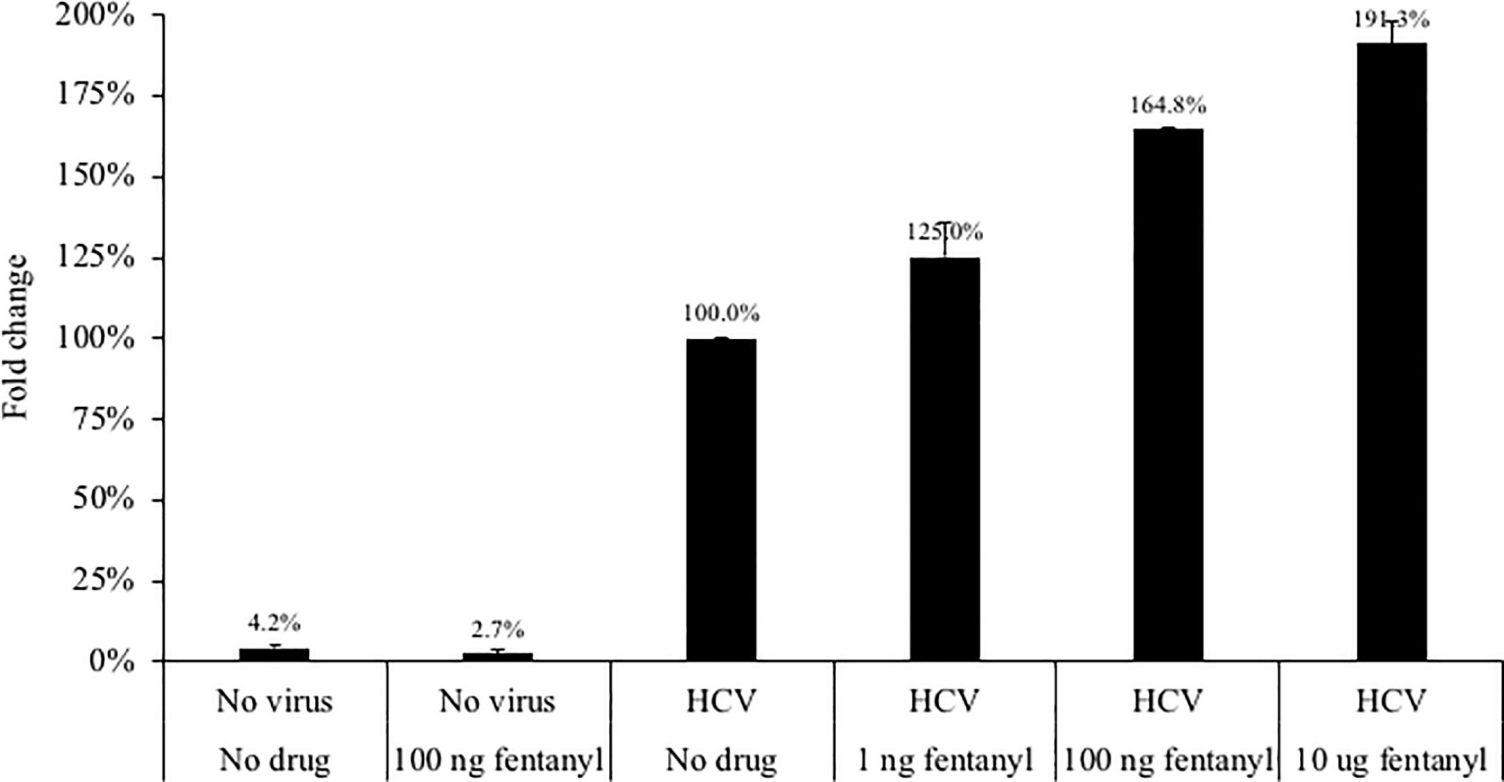
The parental Huh7.5 hepatocyte cell line was seeded at 500,000 cells per well. Infectious HCV (the JFH1 isolate from the Huh7.5_JFH1_ cell line) was added at TCID_50_ of 0.5 for 1 hour. Fentanyl was added to culture medium. After 24 hours of incubation with drug, HCV core protein (ng/mL) was quantified in culture supernatants. ANOVA for dose effect: p < 0.0001.

**Fig 4:**
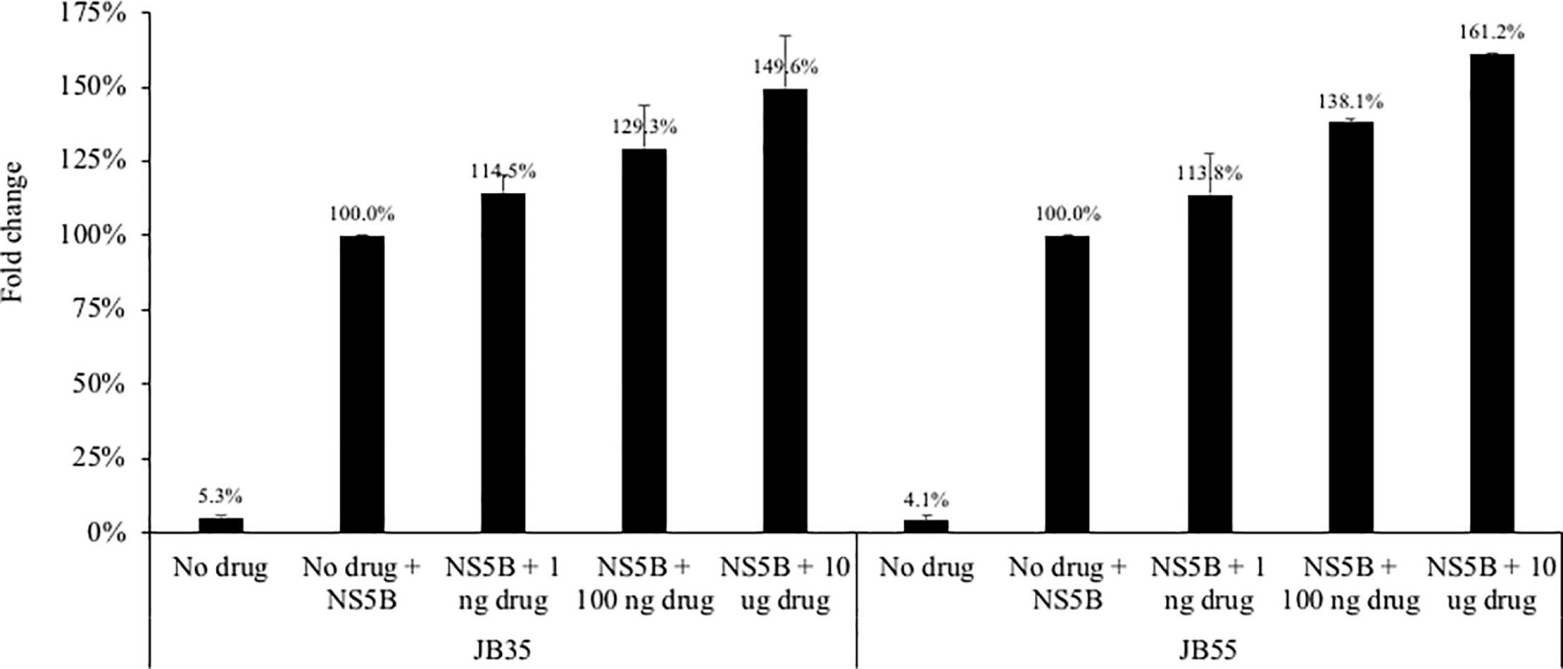
**HCV replicative fitness as measured by patient-specific NS5B shuttle vectors expressing luciferase for patients JB35 (left) and JB55 (right).** ANOVA for dose effect: p = 0.0002 for JB35 and p = 0.0002 for JB55.

The impact of two synthetic opioids–fentanyl and carfentanil–were evaluated utilizing *in vitro* systems that replicate infectious HCV or HBV. As shown in Fig 2, at 24 hours post-drug exposure, low dose (1 ng) fentanyl had no impact on HCV replication in hepatocytes. However, higher doses (100 ng and 10 ug) significantly enhanced HCV replication (187.6% to 193.8% of the no drug condition). Carfentanil increased HCV replication at the low dose of 1 ng (168.1% compared to the no drug condition), although this increase was not maintained at higher doses (107.6% and 66.2% of the no drug condition). A complementary experiment was performed in the parental Huh7.5 cell line that was then exposed to infectious HCV in the presence of fentanyl. As shown in Fig 3, fentanyl resulted in significantly elevated levels of HCV replication in a dose-dependent manner (125.0% to 191.3%) compared to the no drug condition. We next utilized a non-infectious replication model by inserting two distinct patient-derived NS5B sequences inserted into a shuttle vector as described previously [27]. A dose-dependent increase in HCV replicative fitness–as measured by fold change in luciferase activity–was observed for patients JB35 and JB55 in the presence of fentanyl (Fig 4).

**Fig 5:**
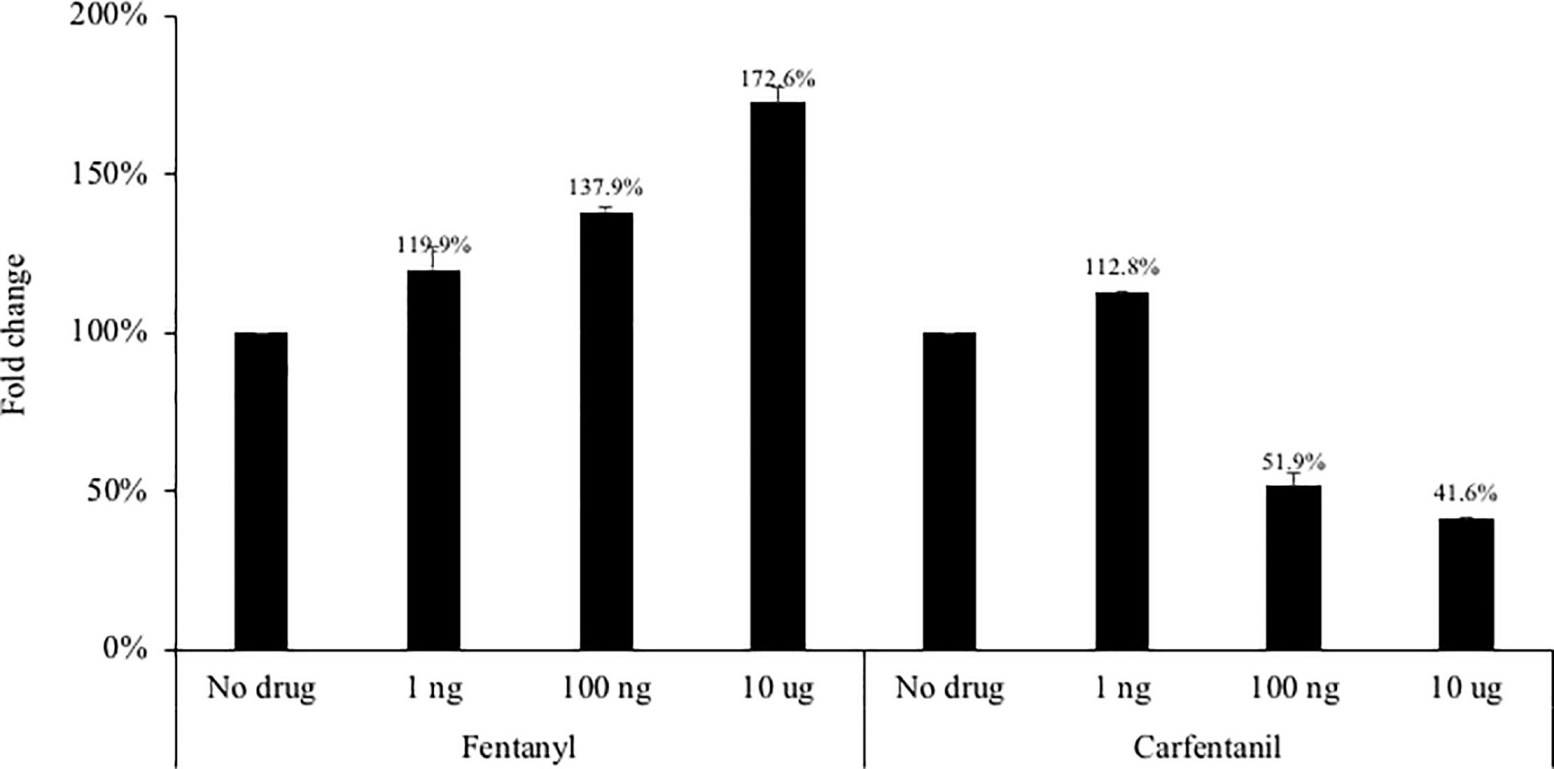
The HepG2.2.15 hepatocyte cell line–which constitutively expresses infectious HBV–was seeded at 500,000 cells per well. Fentanyl or carfentanil was added to culture medium after 24 hours. After 24 hours of incubation with drug, HBsAg protein (ng/mL) was quantified in culture supernatants. ANOVA for dose effect: p = 0.0012 for fentanyl and p = 0.0002 for carfentanil.

To determine if this proviral effect was virus-specific, the HepG2.2.15 hepatocyte cell line which constitutively expresses infectious HBV was evaluated in the presence/absence of fentanyl or carfentanil. As shown in Fig 5, there was a dose-dependent increase in HBV in the presence of fentanyl– 119.9%, 137.9%, and 172.6% at the 3 doses compared to the no drug condition.

Addition of carfentanil led to a modest increase in HBV replication at the low dose (112.8% compared to the no drug condition), although decreased HBV replication was observed at higher doses (51.9% and 41.6% of the no drug condition).

### Opioids increase hepatic apoptosis and decrease cell viability

**Fig 6:**
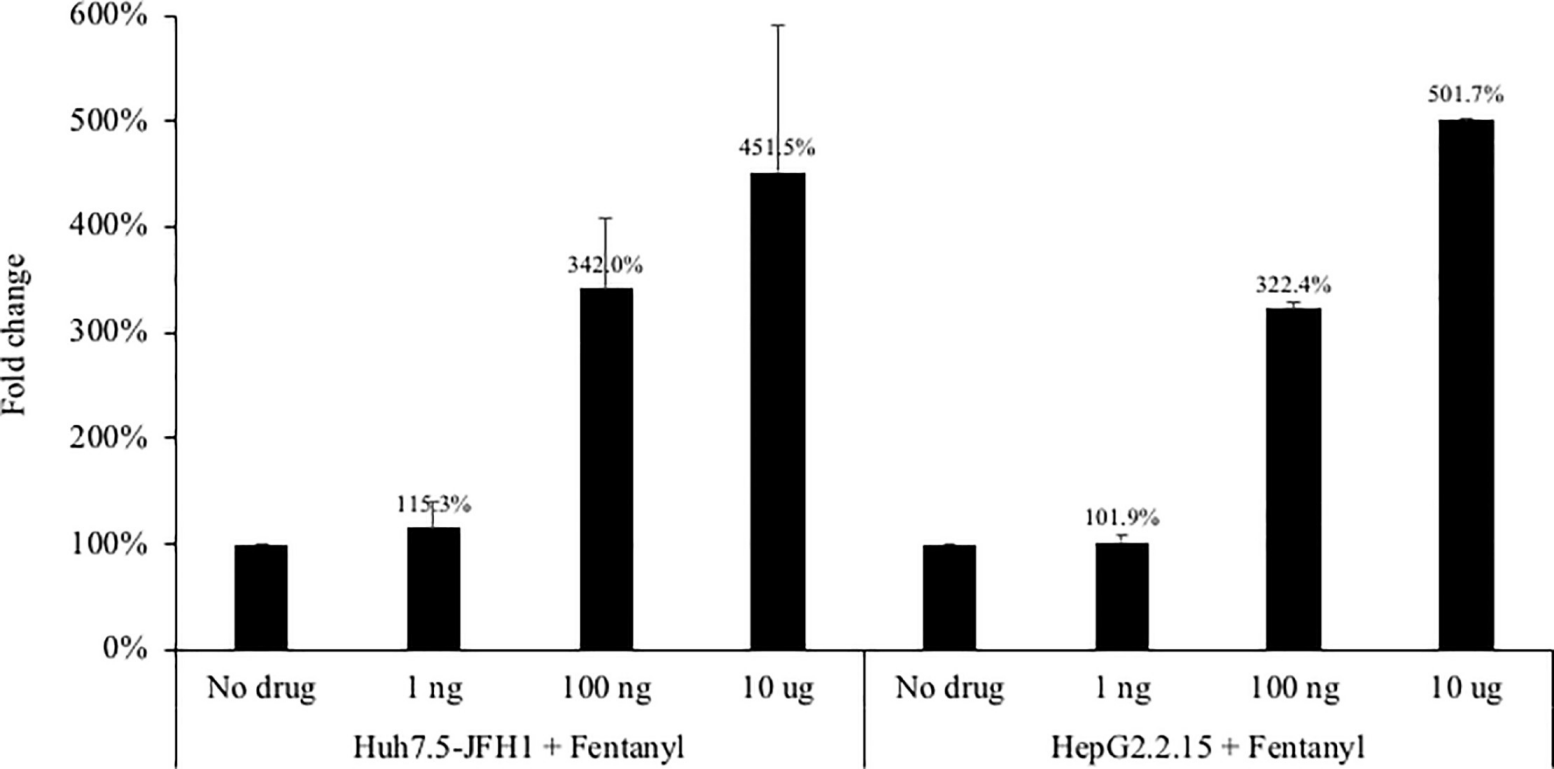
The Huh7.5_JFH1_ or HepG2.2.15 hepatocyte cell lines were seeded at 500,000 cells per well. **Fentanyl was added to culture medium after 24 hours.** After 3 days of incubation with drug, M30 (units/mL) was quantified in culture supernatants. ANOVA for dose effect: p = 0.0018 for Huh7.5_JFH1_ and p = 0.0003 for HepG2.2.15.

The impact of fentanyl on hepatic apoptosis was then evaluated. As shown in Fig 6, exposure to fentanyl resulted in a dose-dependent accumulation of soluble caspase-cleaved keratin 18 (M30)–a product of apoptosis–in two hepatocyte cell lines. At the high fentanyl dose, fold change in apoptosis compared to the no drug condition was 451.5% and 501.7% in the Huh7.5_JFH1_ and HepG2.2.15 cell lines, respectively.

**Fig 7:**
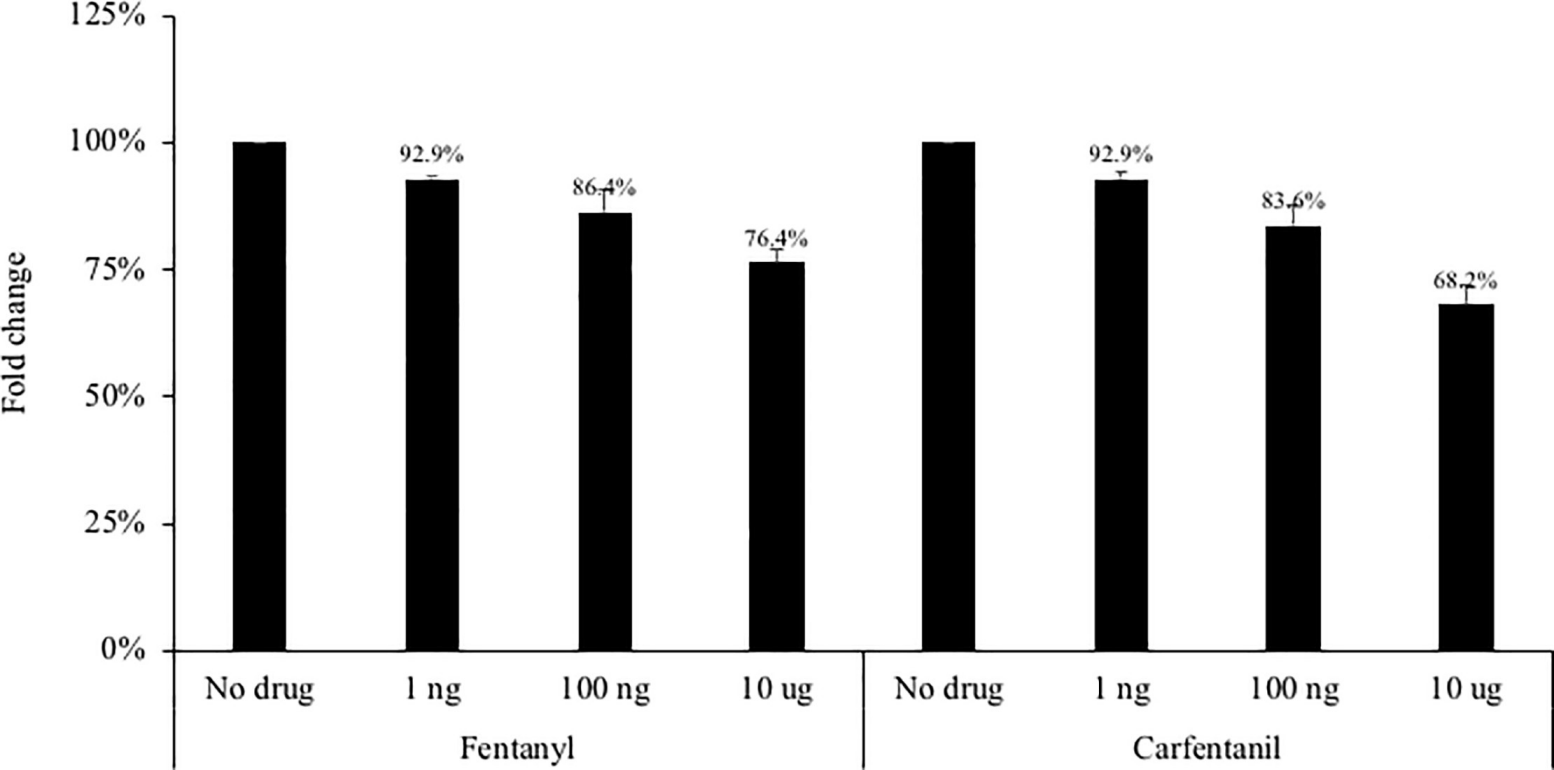
The Huh7.5_JFH1_ hepatocyte cell line was seeded at 500,000 cells per well. **Fentanyl or carfentanil was added to culture medium after 24 hours.** After 24 hours of incubation with drug, the potential toxicity was evaluated by the MTT Cell Proliferation Assay Kit. ANOVA for dose effect: p = 0.044 for fentanyl and p = 0.028 for carfentanil.

As drug exposure may impact cell viability, cell proliferation was quantified for Huh7.5_JFH1_ cells in the presence of fentanyl or carfentanil using the MTT Cell Proliferation Assay (Fig 7). A modest decrease in cell number was observed for the low and medium doses of fentanyl (92.9% to 86.4% compared to the no drug condition) and carfentanil (92.9% to 83.6% compared to the no drug condition). At the high drug dose, decreased cell number was more pronounced (76.4% and 68.2% for fentanyl and carfentanil, respectively).

### Opioids alter the hepatocyte transcriptome

**Fig 8:**
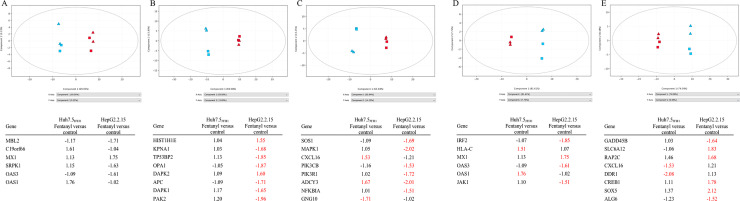
Principle component analysis of mRNA expression data from the Huh7.5_JFH1_ and HepG2.2.15 hepatocyte cell lines in the presence / absence of fentanyl. **(A)** Antiviral, **(B)** cell death, **(C)** chemokine, **(D)** interferon, and **(E)** NFκB signaling genes sorted by cell line (red = Huh7.5_JFH1_ and blue = HepG2.2.15) and drug treatment (square = no fentanyl and triangle = fentanyl treated). Statistically significant fold changes are highlighted in red.

Given the significant impact of fentanyl on viral replication and apoptosis in the Huh7.5_JFH1_ and HepG2.2.15 hepatocyte cell lines, we evaluated further the impact of fentanyl on mRNA expression. After 3 days of incubation with drug, RNAseq identified a number of genes related to apoptosis, viral gene expression, hepatocarcinogenesis, and the NFκB response that were up or down regulated (S1 and S2 Figs). Principal component analysis was then employed for a more detailed exploration of genes related to the antiviral response, cell death, chemokine signaling, the interferon response, and NFκB signaling. As shown in Fig 8, genes clustered by cell type (Huh7.5_JFH1_ versus HepG2.2.15). For most gene families, the data also clustered based on fentanyl treatment status. As shown in Fig 8A, differentially expressed antiviral genes included MBL2, C19orf66, MX1, SRPK1, OAS3, and OAS1, although none achieved statistical significance in either cell line. In contrast, 8 cell death genes (HIST1H1E, KPNA1, TP53BP2, OPA1, DAPK2, APC, DAPK1, and PAK2) were differentially regulated in both cell lines, although these fold changes were only statistically significant in the HepG2.2.15 cell line (Fig 8B). Thus, fentanyl augments cell death processes in Hep2.2.15 but not Huh7.5_JFH1_ cells. Eight genes involved in chemokine signaling were statistically differentially regulated in the Huh7.5_JFH1_ (CXCL16, ADCY3, and GNG10) and/or HepG2.2.15 cell lines (SOS1, MAPK1, PIK3CB, PIK3R1, ADCY3, and NFKB1A) as shown in Fig 8C. Interferon genes were differentially regulated in either the Huh7.5_JFH1_ (HLA-C and OAS1) or HepG2.2.15 (IRF2, MX1, OAS3, and JAK1) cell lines, indicating that fentanyl augments different components of interferon signaling depending upon the cell type (Fig 8D). Finally, 8 genes involved in NFκB signaling were differentially regulated in either the Huh7.5_JFH1_ (CXCL16 and DDR1) or HepG2.2.15 (GADD45B, SLC6A12, RAP2C, CREB1, SOX5, and ALG6) cell lines, suggesting that fentanyl augments different elements of NFκB signaling depending upon the cell type (Fig 8E).

## Discussion

Drugs of abuse are able to promote viral replication and virus-mediated pathology [8]. Currently, the majority of unintentional overdoses in the United States involve fentanyl or fentanyl analogs [3–6]. Nonetheless, it is not known how synthetic opioids may impact viral replication and pathogenesis. As reviewed elsewhere, several distinct opioids promote HIV replication in immune cells [19]. Potential mechanisms include enhanced chemokine receptor expression, inhibition of β chemokines, antagonism of innate antiviral responses, and altered microRNA (miRNA) expression. Even less is known about the potential impact of opioids on viral hepatitis and liver disease. *In vitro* studies demonstrate that morphine, heroin, and methamphetamine can enhance HCV replication [28–32]. *In vivo*, HCV RNA levels are significantly higher among heroin users compared to non-heroin users [30]. Expression of microRNAs that enhance HCV replication are also elevated in heroin users with HCV infection. Morphine treatment inhibits IFNα and increases HCV expression in a dose-dependent manner *in vitro* [29, 31]. Methamphetamine inhibits the antiviral effects of IFNα and promotes viral replication in hepatocytes *in vitro* [28]. Nonetheless, the impact of individual synthetic opioids on HCV expression and dysregulation of host cellular pathways has not been evaluated prior to the current study.

Data presented here demonstrate that fentanyl has a proviral effect on two hepatotropic viruses (HBV and HCV). To our knowledge, this is the first study to show an effect of fentanyl on replication of any virus. As well, despite its transmissibility by IDU, very little is known about the effects of any drugs of abuse–including synthetic opioids–on HBV replication *in vitro* or in HBV-infected individuals with opioid use. While the exact mechanism(s) underlying the proviral effect of fentanyl are not yet known, the RNAseq analyses (Fig 8; S1 and S2 Figs) provide several possibilities, including genes related to the antiviral/interferon response, cell death, chemokine signaling, and NFκB signaling. These findings are supported by previous studies in non-viral systems demonstrating that fentanyl induces apoptosis, alters NFκB signaling, and exerts immunosuppressive effects. For instance, peripheral blood lymphocytes and human umbilical cord mononuclear cells cultured in the presence of fentanyl experience increased apoptosis [33, 34]. Ma *et al*. reported that fentanyl induced apoptosis of activated CD4+ T cells and inhibited their secretion of IFNγ, IL-2, and IL-4 [34]. Others have reported increased apoptosis and/or expression of apoptosis-related genes in non-hepatic cell lines [35–39]. Additionally, several studies have observed suppressed activation of NFκB in the presence of fentanyl [34–36, 39, 40].

Of note, Huh7.5_JFH1_ cells expressed higher levels of mu opioid receptor than the parental, uninfected Huh7.5 cells. Mu opioid receptor gene expression is dependent upon several transcription factors, including activator protein 1 (AP-1) and NFκB and can be altered by multiple cytokines [9, 41–44]. There are limited data on mu opioid receptor expression in the context of viral infection. Chakass *et al*. reported lower mu opioid receptor mRNA expression in liver biopsies and peripheral blood from individuals with untreated chronic HCV infection compared to controls [45]. *In vitro* studies suggest that the HIV proteins may upregulate mu opioid receptor expression [46–48]. However, whether HCV or HBV regulate mu opioid receptor expression in the replication systems evaluated in the current study requires additional investigation.

Interestingly, carfentanil led to increased replication of HBV and HCV replication at the low dose but not at intermediate/high drug doses. Despite similar chemical structures, molecular docking models and radioligand displacement assays highlight distinct binding affinities of fentanyl and carfentanil for the mu opioid receptor [49, 50]. Carfentanil is approved for veterinary use only, and because of its extremely high potency, human studies assessing the metabolism of carfentanil have not been performed to date [26, 51]. Similarly, the impact of carfentanil on cellular gene expression remains poorly explored. Thus, additional investigation is required to determine the exact nature of this drug-specific response in viral replication.

Several caveats to the current study design should be considered. First, *in vitro* drug exposure was short-term. While this may reflect acute drug exposure, it may not adequately reflect long-term drug exposure. Thus, the impact of opioids on cell viability, cell signaling, and/or apoptosis may vary be dose or timing of drug exposure. As well, recreational users likely encounter fentanyl on a more continuous, daily basis that could lead to sustained increases in viral replication. Fentanyl exposure may also co-occur with exposure to other opioids and/or other substances (e.g., methamphetamine, cocaine, etc.). As higher viral loads are often associated with increased likelihood of transmission to others and/or pathogenic consequences, *in vivo* studies conducted in individuals with viral hepatitis and opioid use are needed. Second, while physiologically relevant concentrations of fentanyl are poorly characterized, a limited number of studies suggest that concentrations of 10^-12^M to 10^-4^M are reasonable for the described experiments [52–58]. Third, many fentanyl analogs exist that have similar chemical structure to fentanyl. However, their specific impact on viral replication have not been evaluated here. Fourth, these are *in vitro* interactions that have not be evaluated in clinically relevant populations yet and in vivo studies, as well as complementary evaluation in primary hepatocytes and/or liver organoid systems is warranted. Nonetheless, these findings highlight the need to carefully study virus-opioid interactions for the myriad of opioids and opioid derivatives that are now detected in IDU populations, as well as characterize the various mechanisms in a virus- and drug-dependent manner.

In summary, there is an urgent public health need to address the linked opioid and viral hepatitis epidemics, particularly in regions with high prevalence of opioid use disorder and synthetic opioids. Rigorous characterization of the interactions among HCV and its treatments, fentanyl, and host cells will improve clinical management paradigms for difficult-to-treat populations, facilitate rational public health policies given severely strained resources, and reveal additional pathways for novel target-specific therapeutic interventions.

## Supporting information

S1 Fig RNAseq analysis of genes that are upregulated (red) or downregulated (blue) in the Huh7.5_JFH1_ hepatocyte cell line in the presence / absence of fentanyl.
(TIFF)

S2 Fig RNAseq analysis of genes that are upregulated (red) or downregulated (blue) in the HepG2.2.15 hepatocyte cell line in the presence / absence of fentanyl.
(TIFF)
