# Supplementary figures and images for: The synthetic opioid fentanyl enhances viral replication in vitro
Source: PLoS One. 2021 Apr 14;16(4):e0249581. doi: 10.1371/journal.pone.0249581 (PMC8046189; doi:10.1371/journal.pone.0249581)

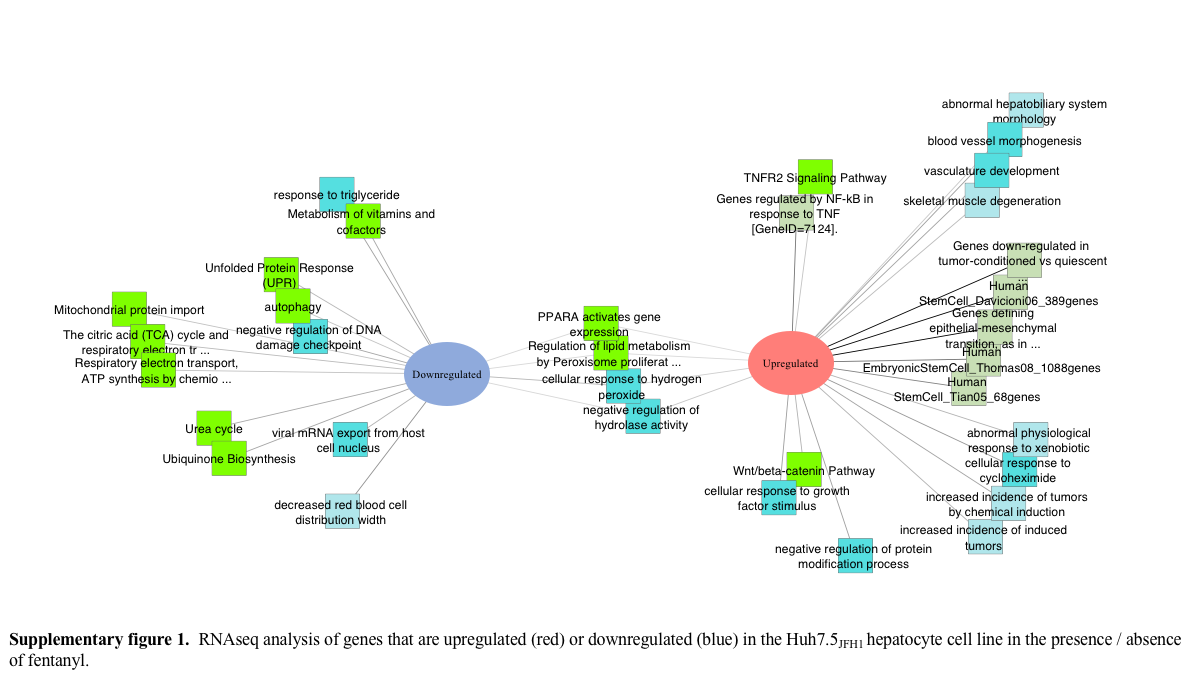

Supplement: S1 Fig — RNAseq analysis of genes that are upregulated (red) or downregulated (blue) in the Huh7.5JFH1 hepatocyte cell line in the presence / absence of fentanyl. (TIFF) [file pone.0249581.s001.tiff]

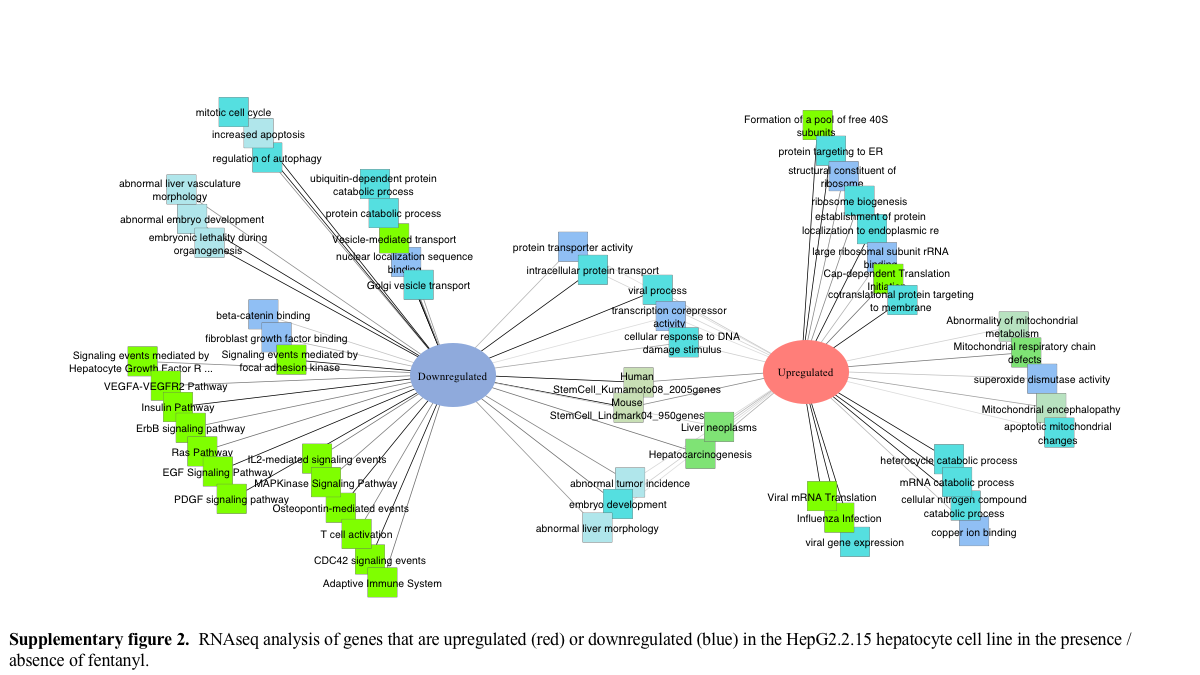

Supplement: S2 Fig — RNAseq analysis of genes that are upregulated (red) or downregulated (blue) in the HepG2.2.15 hepatocyte cell line in the presence / absence of fentanyl. (TIFF) [file pone.0249581.s002.tiff]
